# TM4SF1 Promotes Gemcitabine Resistance of Pancreatic Cancer *In Vitro* and *In Vivo*

**DOI:** 10.1371/journal.pone.0144969

**Published:** 2015-12-28

**Authors:** Jia Cao, Jiachun Yang, Vijaya Ramachandran, Thiruvengadam Arumugam, Defeng Deng, Zhaoshen Li, Leiming Xu, Craig D. Logsdon

**Affiliations:** 1 Department of Gastroenterology, Xinhua Hospital, Shanghai Jiaotong University School of Medicine, Shanghai, 200092, China; 2 Department of Cancer Biology, The University of Texas MD Anderson Cancer Center, Houston, Texas, 77030, United States of America; 3 Department of Gastroenterology, Changhai Hospital, the Second Military Medical University, Shanghai, 200433, China; University of Texas Health Science Ctr, UNITED STATES

## Abstract

**Background:**

TM4SF1 is overexpressed in pancreatic ductal adenocarcinoma (PDAC) and affects the development of this cancer. Also, multidrug resistance (MDR) is generally associated with tumor chemoresistance in pancreatic cancer. However, the correlation between TM4SF1 and MDR remains unknown. This research aims to investigate the effect of TM4SF1 on gemcitabine resistance in PDAC and explore the possible molecular mechanism between TM4SF1 and MDR.

**Methods:**

The expression of TM4SF1 was evaluated in pancreatic cancer cell lines and human pancreatic duct epithelial (HPDE) cell lines by quantitative RT-PCR. TM4SF1 siRNA transfection was carried out using Hiperfect transfection reagent to knock down TM4SF1. The transcripts were analyzed by quantitative RT-PCR, RT-PCR and western blotting for further study. The cell proliferation and apoptosis were obtained to investigate the sensitivity to gemcitabine of pancreatic cancer cells after silencing TM4SF1 *in vitro*. We demonstrated that cell signaling of TM4SF1 mediated chemoresistance in cancer cells by assessing the expression of multidrug resistance (MDR) genes using quantitative RT-PCR. *In vivo*, we used orthotopic pancreatic tumor models to investigate the effect of proliferation after silencing TM4SF1 by a lentivirus-mediated shRNA in MIA PaCa-2 cell lines.

**Results:**

The mRNA expression of TM4SF1 was higher in seven pancreatic cancer cell lines than in HPDE cell lines. In three gemcitabine-sensitive cell lines (L3.6pl, BxPC-3, SU86.86), the expression of TM4SF1 was lower than that in four gemcitabine-resistant cell lines (MIA PaCa-2, PANC-1, Hs766T, AsPC-1). We evaluated that TM4SF1 was a putative target for gemcitabine resistance in pancreatic cancer cells. Using AsPC-1, MIA PaCa-2 and PANC-1, we investigated that TM4SF1 silencing affected cell proliferation and increased the percentages of cell apoptosis mediated by treatment with gemcitabine compared with cells which were treated with negative control. This resistance was associated with the expression of multidrug resistance genes including ABCB1 and ABCC1. *In vivo*, silencing of TM4SF1 in MIA PaCa-2 cell lines increased the effectiveness of gemcitabine-based treatment in orthotopic pancreatic tumor models evaluated using noninvasive bioluminescent imaging.

**Conclusion:**

These findings suggest that TM4SF1 is a surface membrane antigen that is highly expressed in pancreatic cancer cells and increases the chemoresistance to gemcitabine. Thus, TM4SF1 may be a promising target to overcome the chemoresistance of pancreatic cancer.

## Introduction

Pancreatic ductal adenocarcinoma (PDAC) is the most aggressive human malignancy with a high mortality rate [1]. The average 5-year survival rate of patients diagnosed with PDAC is 7.1% and the median survival duration is shorter than 6 months [2, 3]. Since lacking of early specific symptoms, the majority of patients are diagnosed in late stages. Chemotherapy using gemcitabine is considered as a first-line treatment for locally advanced and metastatic pancreatic cancer to prolong the survival time and improve the quality of patients’ life [4]. However, the high rate of resistance to gemcitabine decreases the anti-tumor efficacy [5]. Thus, effective chemotherapeutic methods are still urgently needed to improve the outcome for this cancer.

Multidrug resistance (MDR) is characterized by cross-resistance to chemotherapeutic drugs with target sites [6]. The major mechanisms of gemcitabine chemoresistance are related with drug uptake, metabolism and action [4]. One of the common reasons to result in MDR in cancer cells is via overexpressing the adenine triphosphate (ATP)-binding cassette (ABC) transporters. These transporters contribute to the MDR by inducing the antiapoptotic machinery, increasing the intracellular drug efflux and reducing the drug concentrations [7]. Recently, studies have shown that gemcitabine resistance in pancreatic cancer cells is associated with the expression of MDR [8,9]. Therefore, it is important to inhibit these transporters to restore the sensitivity to chemotherapeutic resistance in pancreatic cancer.

TM4SF1 is a member of the four-transmembrane-domain family with a tetraspanin topology and sequence homology with IL-TMP, TM4SF5, L6D, OCTM4, and TCCE518. The expression levels of TM4SF1 are increased in various human epithelial carcinomas including lung, breast, colon, ovarian, prostate and renal carcinomas [10–12]. It plays a critical role in regulating tumor angiogenesis, motility, migration and invasion [13, 14]. Importantly, recently study has shown that TM4SF1-targeting Antibody Drug Conjugates represent a promising therapeutic agent against tumor cells and the tumor vasculature [15]. Thus, the goal of this study was to investigate whether TM4SF1-overexpressing pancreatic cancer cell lines are involving in gemcitabine resistance and to delineate the mechanism.

In the present study, we sought to determine whether TM4SF1 could confer pancreatic cancer with the ability to gemcitabine resistance.We found that TM4SF1 was highly expressed in gemcitabine-resistance pancreatic cancer cell lines. Silencing of TM4SF1 increased the gemcitabine sensitivity of chemoresistant cells and decreased the expression of ABCB1 and ABCC1 *in vitro*. *In vivo*, cells lacking TM4SF1 had reduced pancreatic tumor growth and increased responsiveness to treatments with gemcitabine. These results suggest that TM4SF1 should be investigated further as a potential target for pancreatic cancer therapy.

## Materials and Methods

### Drugs and Reagents

Gemcitabine was purchased from Eli Lilly and Co (Indianapolis, IN). All reagents were provided by Sigma-Aldrich (St. Louis, MO, USA), unless specified below.

### Pancreatic cancer cell lines and culture conditions

The human pancreatic cancer cell lines HS766T, MIA PaCa-2, PANC-1, AsPC-1, BxPC-3, L3.6PL and SU.86.86 were obtained from the American Type Culture Collection (ATCC). BxPC-3 and AsPC-1 cells were routinely cultured in RPMI-1640 medium supplemented with 10% fetal bovine serum in a 37°C incubator in a humidified atmosphere of 5% CO2, whereas all other pancreatic cancer cell lines grew in Dulbecco’s modified Eagle’s medium, Human pancreatic duct epithelial (HPDE) cells [16] provided by Dr. Tsao (University of Toronto) were cultured in keratinocyte serum-free medium. The cell lines were obtained from ATCC and were authenticated by short tandem repeat profiling and passaged in our laboratory for fewer than 6 months after receipt or resuscitation.

### Quantitative real-time reverse transcription PCR analysis

Total RNA was isolated from pancreatic cancer cell lines and HPDE, and the quality of the RNA was determined as described previously [17]. The RNA was used for quantitative reverse transcription-polymerase chain reaction analysis. Real-time quantitative reverse transcription-polymerase chain reaction was performed with SYBR green as the tag using an iCycler. (Bio-Rad).Sequence of the primers is available upon request.

### Reverse transcription PCR analysis

PCR was carried out using a PCR Kit (Promega) with the following amplification program:pre-denaturation for 3 min at 94°C, denaturation for 30 sec at 94°C, annealing for 30 sec at 60°C,extension for 30 sec at 72°C, and a final elongation step at 72°C for 10min. PCR was performed for 30 cycles. After PCR, 5 μl of PCR products were run on a 1% agarose gel and visualized by relative pixel densitometry.

### Transient transfection of small interfering RNA

AsPC-1, MIA PaCa-2, and PANC-1 cells were plated on 100-mm dishes and transiently transfected with control small interfering RNA (siRNA [siControl]) and TM4SF1 siRNA (siTM4SF1) at final concentrations of 10 nmol/L with Hiperfect transfection reagent (QIAGEN). The siRNA sequence targeting TM4SF1 was AAGGACCACTATGTCTTGATT. mRNAs were isolated from AsPC-1, MIA PaCa-2, and PANC-1 cells for quantitative RT-PCR after 48 hours.

### Western blotting analysis

Protein was extracted with RIPA buffer supplemented with 1% PMSF for 30 min. Each extract containing 50μg protein was separated by 10% SDS-polyacrylamide gel electrophoresis and transferred to polyvinylidene difluoride (PVDF) membranes. The membranes were blocked in 5% non-fat dry milk for 3h then incubated with rabbit anti-TM4SF1 (ABCAM, 1:1000 dilution) at 4°C overnight. Membranes were incubated with horseradish peroxidase-conjugated goat anti-rabbit IgG antibody for 2h at room temperature. Blots were detected using the ECL (Millipore) and exposed to X-ray film to visualize the images.

### Development of stable short hairpin RNA-expressing cell lines

Silencing of TM4SF1 in pancreatic cancer cell lines was achieved using lentiviral infection. Lentiviral plasmid vectors containing-control and TM4SF1 short hairpin RNA (shRNA; Open Biosystems) were co-transfected with packaging vectors and the lentivirus was produced in 293T cells via calcium transfection as described previously [17].TM4SF1 shRNA-containing vectors were titrated, and MIA PaCa-2 was infected with 25 μL of viral supernatant/per milliliter of medium mixed with polybrene (4 μg/mL medium). Cells were then selected according to their resistance to puromycin (1–3 μg/mL). Stable pancreatic cancer cell line was developed thereafter.

### Proliferation assay

We used MTS assay to examine the effects of gemcitabine after silencing TM4SF1 on the proliferation of AsPC-1, Mia PaCa-2 and PANC-1 cell lines. Cells growing on a 6-well plate were treated with control siRNA or siTM4SF1. 24h after transfection, cells were collected and 1500 cells were plated into 96-well plates. After cells were treated with gemcitabine for 48h, 15μL of MTS solution was added to each well, incubated at 37°C for 2h. Cell numbers were estimated using the photometric reading, as described previously [18,19].

### Apoptosis study using fluorescence-activated cell sorting analysis *in vitro*

In previous study, we found the pancreatic cancer cell lines AsPC-1, MIA PaCa-2 and PANC-1 were resistant to gemcitabine *in vitro* [19]. Standard propidium iodide staining of pancreatic cancer cells using the hypotonic lysis method was used for apoptosis studies with fluorescence-activated cell sorting (FACS). AsPC-1, MIA PaCa-2 and PANC-1 cells transiently transfected with siControl or siTM4SF1 for 24 hours were seeded in six-well plates. Apoptosis was induced in the cells by treating them with gemcitabine (1, 5 or 10 μmol/L) for 48 or 72 hours. The cells were then collected via trypsinization, fixed with 70% cold ethanol, mixed with 500 μL of a hypotonic solution (0.1% sodium citrate, 0.1% Triton X-100, 20 μg/ml RNase, and 50 μg/ml propidium iodide), incubated for 30-minutes and analyzed via flow cytometry using an EPICS-XO system (Beckman Coulter). Cells undergoing apoptosis owing to DNA fragmentation were detected as the population of cells with sub-G1 DNA content.

### Animals and Tumor growth study *in vivo*

This study was carried out in strict accordance with the guidelines for the Care and Use of Laboratory Animals from the National Institutes of Health. The use of mice was approved by Institutional Animal Care and Use Committee of MD Anderson Cancer Center.

MIA PaCa-2 cells were modified to stably express a firefly luciferase gene via lentiviral transfection [20]. The tumorigenic ability of TM4SF1 shRNA-silenced cells was compared with that of control shRNA-transfected cells in orthotopic pancreatic tumors that formed in 5-week-old male athymic nude nu/nu mice. 28 nude nu/nu mice were divided into four groups (group 1, shCtrl; group 2, shCtrl+GEM; group 3, shTM4SF1; group 4, shTM4SF1+GEM). All animals were given sterile food and autoclaved water on a daily 12-hour light/12-hour dark cycle. MIA PaCa-2 cells were stably transfected with shTM4SF1 or control shRNA. These cells then grew to 80% confluence, and were harvested via trypsinization, washed once in PBS, and resuspended to a final concentration of 5×10^6^ cells/ml. Cell suspensions (50μl each) were injected into the pancreases of seven mice per test group. After one week, these mice were treated with or without gemcitabine (100 mg/kg body weight). Tumor growth was assessed using bioluminescent imaging and the survival of animals was recorded. Mice were monitored every week for any symptoms and signs. After 7 weeks, two mice had the signs of dyspnea and abnormal movement and one of the tumors was exceeded 15% body mass. All mice were sacrificed in isoflurane anesthesia followed by cervical dislocation and each mouse’s pancreas was removed. Also, the tumors were weighed.

The bioluminescent imaging was conducted using a cryogenically cooled imaging system coupled with a data acquisition computer running the Living Image software program (Xenogen). Before imaging, animals were anesthetized in an acrylic chamber with a 1.5% isoflurane/air mixture and injected intraperitoneally with 15 mg/mL Luciferin potassium salt in PBS at a concentration of 150 mg/kg body weight. Digital graysclae animal images were acquired, which was followed by acquisition and overlay of a pseudocolor image representing the spatial distribution of detected photons emerging from active luciferase in each animal. Signal intensity was quantified as the sum of all detected photons within the region of interest per second.

### Cell proliferation study using a proliferating cell nuclear antigen assay *in vivo*

Analysis of pancreatic cancer cell proliferation in tumor tissue was performed using a commercially available proliferating cell nuclear antigen (PCNA) kit (Jackson ImmunoResearch) as described in detail previously [21]. Paraffin-embedded sections of tumor tissue treated with and without gemcitabine were analyzed for proliferation. Immunohistochemical stains of the sections were analyzed using an Olympus microscope. Images of the sections were captured using a chilled charge-coupled device camera (Photometrics) and the Smart Capture software program (Digital Scientific). To quantify the proliferation events, the staining results were evaluated by a pathological scientist (Dr. Henry Wang).

### Statistical analysis

All of the *in vitro* experiments were conducted in triplicate and carried out three or more times. Data are presented as the means from independent experiments (±standard error [SE]). Statistically significant differences were determined using Kruskal-Wallis or two-tailed unpaired Student t-test, Dunnett multiple comparison test and Mann-Whitney U test. P levels less than 0.05 were considered statistically significant.

## Results

### TM4SF1 is highly expressed in pancreatic cancer cell lines

**Fig 1:**
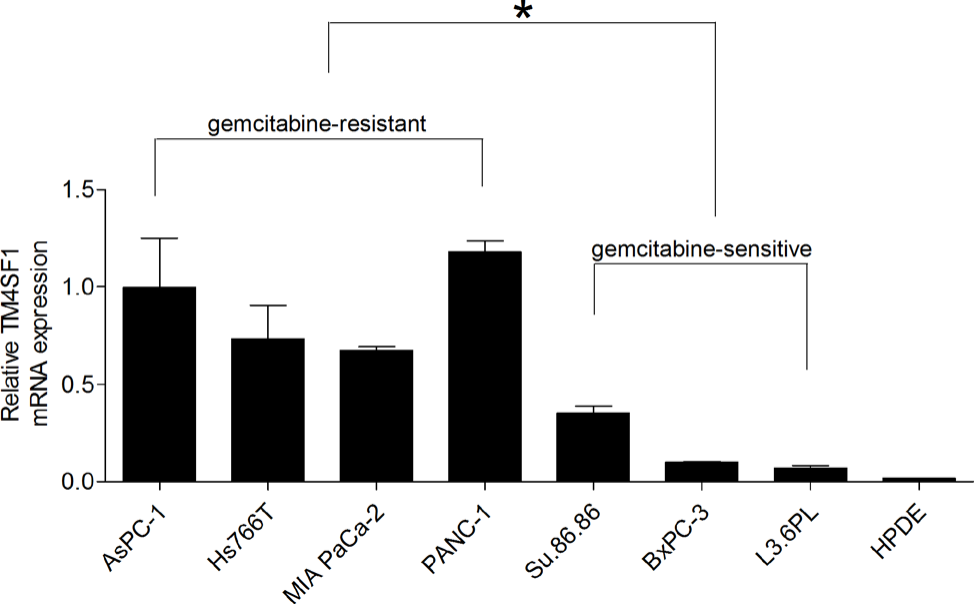
Expression of TM4SF1 in pancreatic cancer cell lines. Quantitative RT-PCR analysis resulted regarding the expression of TM4SF1 mRNA in human pancreatic cancer cell lines.TM4SF1 mRNA was more highly expressed in the cancer cell lines than in HPDE cells. The mRNA expression of TM4SF1 was lower in three gemcitabine-sensitive cell lines (L3.6pl, BxPC-3, SU86.86) than that in four gemcitabine-resistant cell lines (MIA PaCa-2, PANC-1, Hs766T, AsPC-1).

In previous profiling studies, TM4SF1 mRNA expression was elevated in pancreatic tumors and cancer cell lines [22]. And the study found that four cell lines (BxPC-3, SU86.86, CFPAC-1, L3.6pl) were sensitive and five cell lines (PANC-1, Hs766T, MIA PaCa-2, AsPC-1, Mpanc96) were resistant to gemcitabine based on 50% growth inhibition [19]. We further investigated TM4SF1 mRNA expression in seven pancreatic cancer cell lines and HPDE cells. Quantitative RT-PCR analysis indicated that all pancreatic cancer cell lines expressed of TM4SF1 to a greater extent than did the non-transformed HPDE cells. The mRNA expression of TM4SF1 in four gemcitabine-sensitive cell lines (MIA PaCa-2, PANC-1, Hs766T, AsPC-1)was higher than that in three gemcitabine-resistant cell lines (L3.6pl, BxPC-3, SU86.86) (Fig 1).

### TM4SF1 silencing enhances the sensitivity of gemcitabine

**Fig 2:**
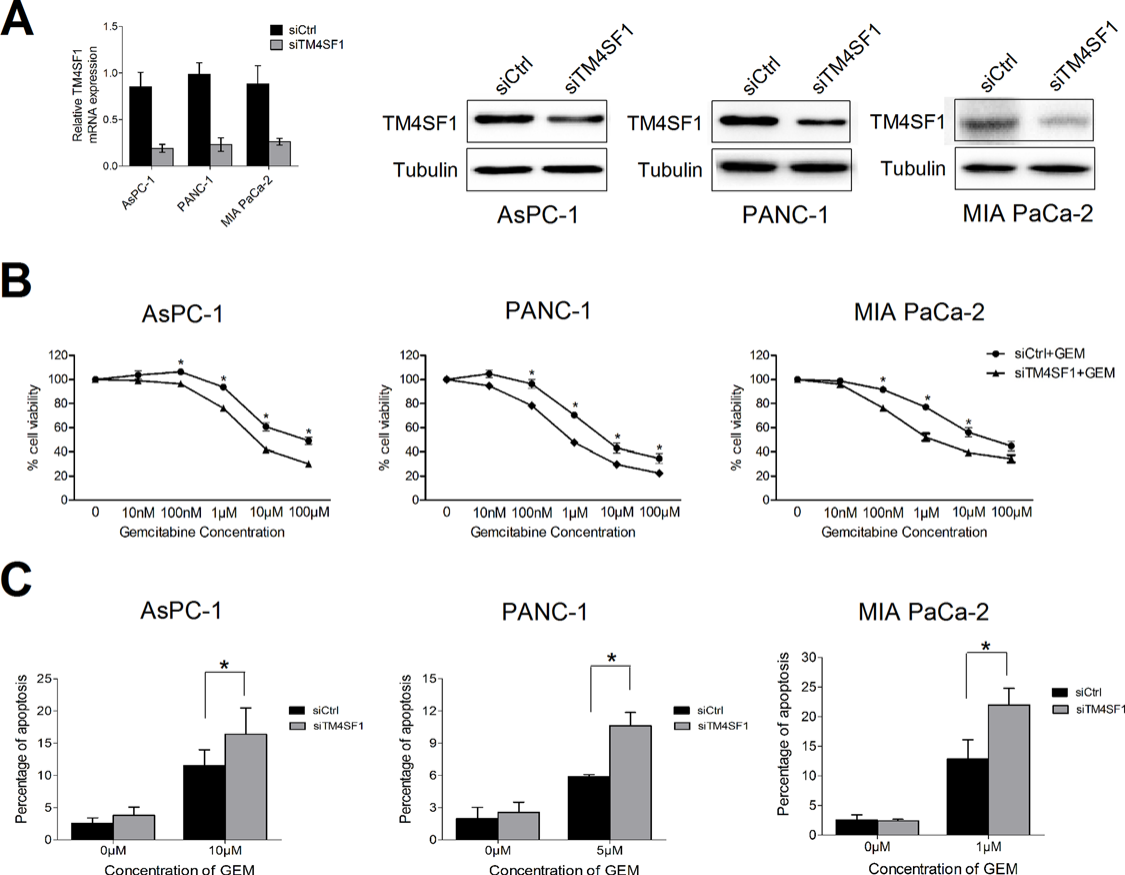
For proliferation assays and apoptosis studies, AsPC-1, MIA PaCa-2, and PANC-1 cells were transfected with siControl or siTM4SF1 and then treated with or without gemcitabine. (A) Quantitative RT-PCR and western blotting demonstrated the silencing of TM4SF1 in AsPC-1, MIA PaCa-2 and PANC-1 cell lines. The cell lines were transiently transfected with siControl or siTM4SF1, and mRNA was isolated from them after 48 hours and protein was isolated after 72 hours. (B) AsPC-1, PANC-1 and MIA PaCa-2 cells were incubated with various concentrations of gemcitabine, respectively. The results of MTS assay showed that the proliferation ability of siTM4SF1 cells was lower than that of siControl cells after treating with gemcitabine. (C) After 72 hours, the percentage of cells with sub-G0/G1 DNA content was identified via FACS. TM4SF1 silencing resulted in increased sensitivity of the cells to treatment with gemcitabine, resulting in increased apoptosis rates. (Columns, means for three experiments; bars, standard deviation. *P < 0.05 versus control.).

To investigate the functional role of TM4SF1 in pancreatic cancer cells, we transiently transfected AsPC-1, MIA PaCa-2, and PANC-1 cells with siControl and siTM4SF1 and confirmed silencing of TM4SF1 in the cells using quantitative RT-PCR, RT-PCR and western blotting (Fig 2A and S1 Fig).To evaluate the effects of TM4SF1 on cell survival and resistance to chemotherapy, we treated MIA PaCa-2, PANC-1 and AsPC-1 cells, all of which express high levels of TM4SF1 natively, with various concentration of gemcitabine in the presence of either siControl or siTM4SF1. MTS assay results showed that proliferation capacity was lower in siTM4SF1 cells than that in siControl cells after treating with gemcitabine (Fig 2B). Also, silencing of TM4SF1 increased the apoptotic response to treatment with gemcitabine with previous concentration(1, 5 or 10μmol/L) in all three cell lines [19] (Fig 2C).

### TM4SF1 regulates the expression of multidrug resistance genes in pancreatic cancer cells *in vitro*

**Fig 3:**
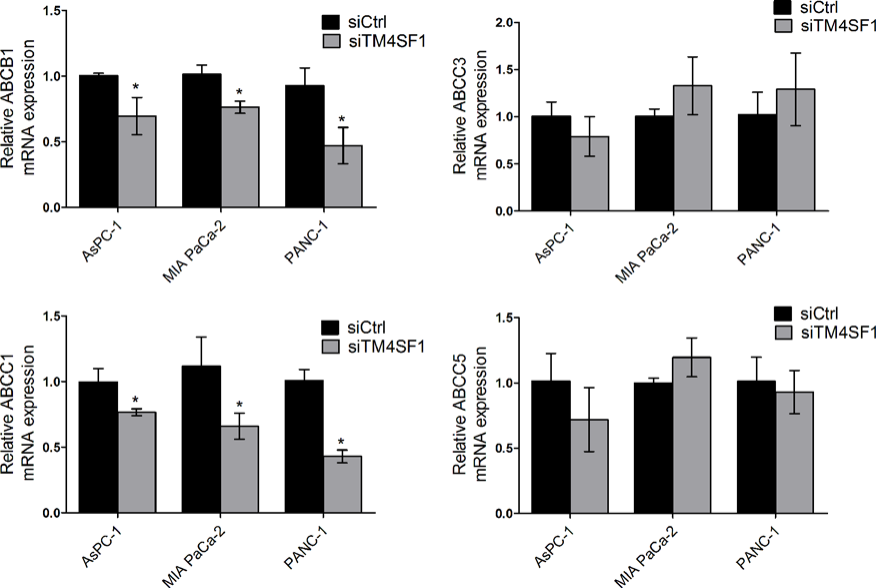
TM4SF1-silencing pancreatic cancer cells mediated the expression of MDR genes. Quantitative RT-PCR data showing fold changes in the mRNA level of ABCB1 and ABCC1 genes after silencing TM4SF1 in AsPC-1, MIA PaCa-2 and PANC-1 cell lines.

Multidrug resistance (MDR) genes contribute to gemcitabine sensitivity in pancreatic cancer cells. The ABCB1, ABCC1, ABCC3 and ABCC5 are well-characterized MDR genes. In AsPC-1, MIA PaCa-2 and PANC-1 cell lines, the mRNA expressions of ABCB1 and ABCC1 were significantly decreased after silencing TM4SF1 using quantitative RT-PCR (Fig 3).

### TM4SF1 silencing increases the sensitivity of pancreatic cancer cells to gemcitabine-induced growth *in vivo*

**Fig 4:**
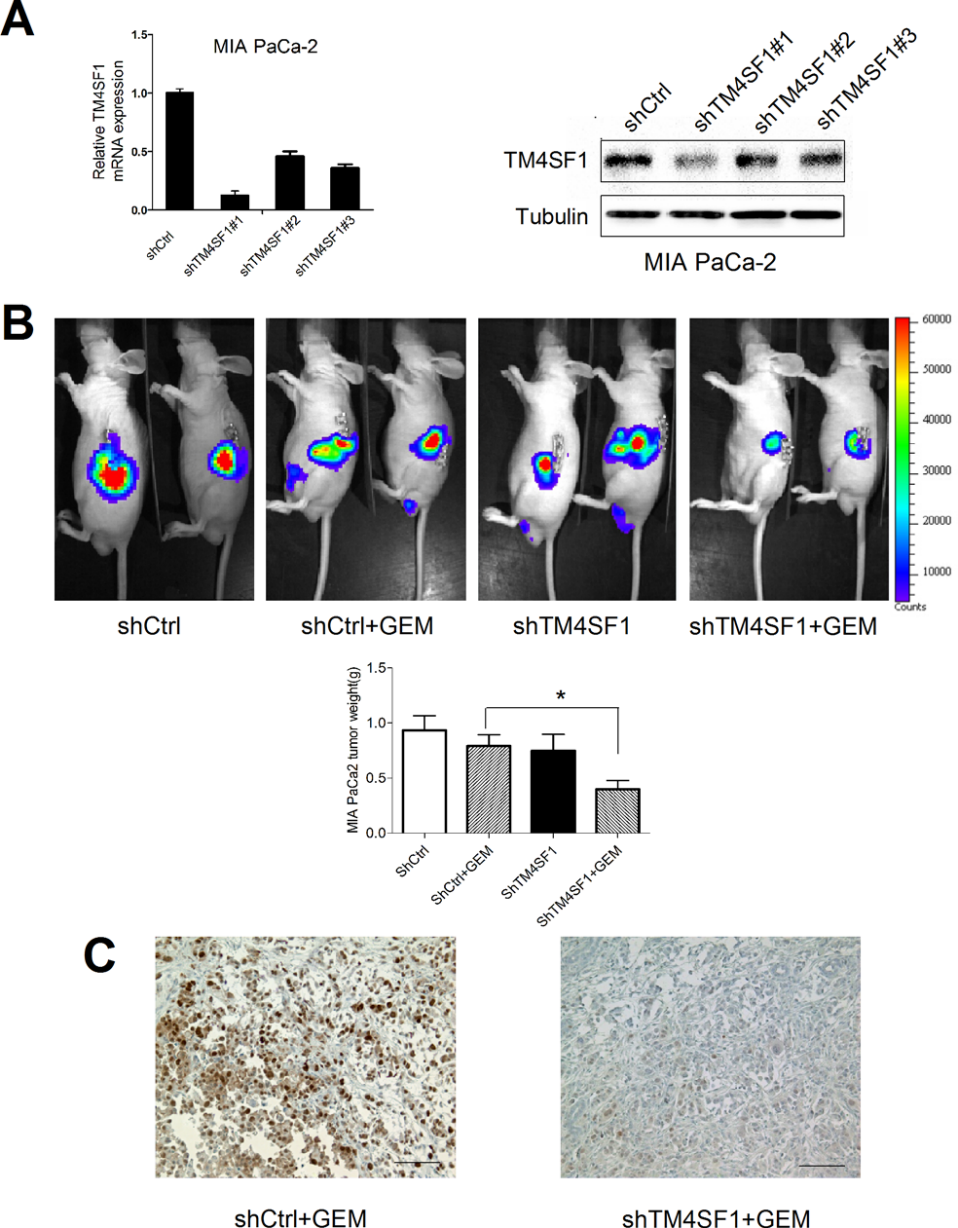
Increased sensitivity of pancreatic cancer cells to treatment with gemcitabine *in vivo* resulting from TM4SF1 silencing. Quantitative RT-PCR and western blotting showed the silencing of TM4SF1 in MIA PaCa-2 stably transfected with shControl or shTM4SF1. (B) Tumors induced by MIA PaCa-2 cells stably expressing shControl or shTM4SF1 and expressing the luciferase gene developed in nude mice. At the end of the experiment, the animals were analyzed using bioluminescent imaging. Weights of tumors induced by MIA PaCa-2 cells stably expressing shRNA with or without gemcitabine (100mg/kg) treatment in nude mice.The tumor weights (g) of shCtrl, shCtrl+GEM, shTM4SF1 and shTM4SF1+GEM were 0.934±0.132, 0.792±0.101, 0.750±0.149 and 0.398±0.080. TM4SF1 silencing by gemcitabine resulted in markedly lower tumor weights than in the control mice (*P < 0.05). (C) *In vivo* proliferation was measured by PCNA assay on paraffin sections from shControl and shTM4SF1 animals treated with gemcitabine. A representative image at a magnification of ×200 indicating cell proliferation is shown.

To further analyze the influence of TM4SF1 *in vivo*, we transfected MIA PaCa-2 having relatively high levels of endogenous TM4SF1 expression with shRNA constructs to stably reduce TM4SF1 expression. We verified shRNA silencing using quantitative RT-PCR and western blotting and observed that TM4SF1 mRNA expression decreased by more than 80% with shTM4SF1#1 (Fig 4A). Next, we analyzed the effects of stably silencing TM4SF1 on pancreatic tumors developed in immuno-deficient mice. Orthotopic tumors were developed with MIA PaCa-2 cells stably expressing shControl or shTM4SF1 and were monitored using noninvasive bioluminescent imaging. Cells lacking TM4SF1 developed tumors to a similar extent as controls. However, gemcitabine treatment had almost no effect on the shControl-transfected groups of these highly gemcitabine-resistant cells, but after silencing TM4SF1 in these cells, gemcitabine-based treatment resulted in markedly lower tumor volumes (Fig 4B). Supporting the sensitization of the MIA PaCa-2 cells to treatment with gemcitabine by silencing of TM4SF1, PCNA analysis (Fig 4C) indicated markedly lower proliferation of tissues with silenced TM4SF1 and treated with gemcitabine than that of all other tissues (Table 1).

**Table 1:** PCNA staining in tumors.

Group	−	±	+	++	+++
shControl	0	0	0	2	5
shControl+GEM	0	0	0	2	5
shTM4SF1	0	0	3	1	3
shTM4SF1+GEM*	1	2	3	1	0

TM4SF1 silencing combined with gemcitabine treatment reduces the proliferation *in vivo*

Tumors were developed in nude mice with MIA PaCa-2 cells stably expressing shControl or shTM4SF1, and these animals were treated with and without gemcitabine. At the end of the experiment, proliferation was measured by PCNA assay on paraffin sections. Blind to grade the PCNA staining -~+++. TM4SF1 silencing in combination with gemcitabine significantly reduced the cell proliferation.

*P < 0.05 versus control, Mann-Whitney U test, P = 0.002

## Discussion

Both metastasis and chemoresistance were associated with reduced survival of cancer patients. Researches investigated that TM4SF1 as a tetraspanin traversing the membrane may be associated with the tetraspanin-enriched microdomanin (TERM) on the plasma membrane, playing roles in organizing the integrity of membrane receptors including integrins to affect cell adhesion, migration and metastasis. TM4SF1 was also located on intracellular vesicles and was targeted to late endocytic organelles through a biosynthetic pathway to influence cell motility [13, 23]. The L6 antibody-based immunotherapy achieved partial remissions in patients with metastatic breast cancer. Several researches showed that TM4SF1 was overexpressed in human tumor vascular endothelial cells and TM4SF1-antibody was selectively targeted to tumor blood vessel endothelial cells and tumor cells with little toxicity. TM4SF1 affected vascular maturation and interacted with integrins to induce endothelial cells migration and cell-cell interactions [24–27]. Jaminet’s group investigated novel methods for joint antibodies such as 8G4 with cytotoxic drugs may be expected to treat with tumor and tumor angiogenesis [28]. The previous research revealed that TM4SF1 was involved in the development of pancreatic cancer and inhibited cells migration and invasion [29, 30]. However, it was unknown whether TM4SF1 contributed to chemoresistance of gemcitabine in PDAC.

Therefore, we focused on the role of TM4SF1 in pancreatic adenocarcinoma by observing the effects of silencing this gene on the chemotherapeutic agent *in vitro* and *in vivo*. In our study, we provided the evidence that TM4SF1 was more highly expressed in pancreatic cell lines than in normal pancreatic epithelial (HPDE) cells and gemcitabine resistance in pancreatic cancer cells could result from the expression of TM4SF1. Silencing TM4SF1 decreased the cell proliferation capacity, enhanced the apoptosis cells and partially reversed the chemoresistance of gemcitabine in AsPC-1, MIA PaCa-2, and PANC-1 cell lines. Previous work investigated that chemoresistance in pancreatic cancer cells was via regulating the expressions of MDR genes including ABCB1, ABCC1, ABCC3 and ABCC5 [9]. So, we evaluated whether TM4SF1 induced the expressions of MDR genes to affect gemcitabine sensitivity in pancreatic cancer cells. After silencing TM4SF1, the expressions of two ABC transporters (ABCB1, ABCC1) genes decreased significantly in AsPC-1, MIA PaCa-2, and PANC-1 cells, which provided evidence that TM4SF1 may be correlated with MDR genes to regulate gemcitabine resistance. *In vivo*, orthotopic tumors were developed with MIA PaCa-2 cells expressing shControl or shTM4SF1 in the immuno-deficient mice, which were monitored using noninvasive bioluminescent imaging. We found that silencing TM4SF1 could enhance the sensitivity of MIA PaCa-2 cells to gemcitabine-induced growth. Also, PCNA analysis showed that the proliferation of tissues with both silenced TM4SF1 and treated with gemcitabine was lower than that of all other tissues. These findings suggested that TM4SF1 could be a potential chemoresistance target for pancreatic cancer.

Here, we first provided evidence that TM4SF1 mediated gemcitabine resistance via regulating MDR genes expression. Studies found that MDR genes induced cancer cells to acquire chemoresistance via affecting the efflux of anticancer drugs from cells and decreasing intracellular drug concentrations [31]. Therefore, more efforts should be made to overcome MDR and to investigate new molecular therapies such as gene therapy. In pancreatic cancer, researches showed that MDR proteins were frequently expressed and affected chemotherapy sensitivity and benefit in patients with advanced pancreatic cancer [32–34]. Another research suggested that gefitinib reversed the expression of MDR and restored chemosensitivity of resistance in BxPC-3 cell lines via RAF1/ERK signaling pathway [35]. MUC1 was a membrane-bound glycoprotein which overexpressed in PDAC to increase chemoresistance of gemcitabine and etoposide. In mechanism, MUC1 induced the expression of ABCC1 via an AKT-independent signaling and particularly it was directly related with the promoter region of the ABCC1 gene, indicating that MUC1 may act as a transcriptional regulator [9].Further regulatory mechanism of TM4SF1 and MDR could be studied on resistance to chemotherapeutic drugs in PDAC.

In addition, TM4SF5, another member of the four-transmembrane-domain family, was observed to be highly expressed in human liver cancer tissues and cells. Studies in that cancer suggested that TM4SF5 could enhance p27^Kip1^ expression and phosphorylation and lead to epithelial-mesenchymal transition (EMT) to mediate tumorigenesis, metastasis and gefitinib resistance [36–39]. Further studies will be required to understand the relationships between the different members of the four-transmembrane-domain family.

In conclusion, our study described that silencing of TM4SF1 sensitized pancreatic cancer cells via downregulating ABCB1 and ABCC1 to kill by treatment with gemcitabine may be of particular translational significance. These data suggested that down-regulation of TM4SF1 expression may be of clinical benefit in pancreatic cancer treatment. Future efforts should be devoted to understanding the mechanisms and pathways involved in the effects of TM4SF1 expression on chemoresistance of pancreatic cancer.

## Supporting Information

S1 Fig RT-PCR analysis results demonstrating the silencing of TM4SF1 in AsPC-1, MIA PaCa-2 and PANC-1 cell lines. The cell lines were transiently transfected with siControl or siTM4SF1, and mRNA was isolated from them after 48 hours.
(PDF)
