# Supplementary figures and images for: TM4SF1 Promotes Gemcitabine Resistance of Pancreatic Cancer In Vitro and In Vivo
Source: PLoS One. 2015 Dec 28;10(12):e0144969. doi: 10.1371/journal.pone.0144969 (PMC4692438; doi:10.1371/journal.pone.0144969)

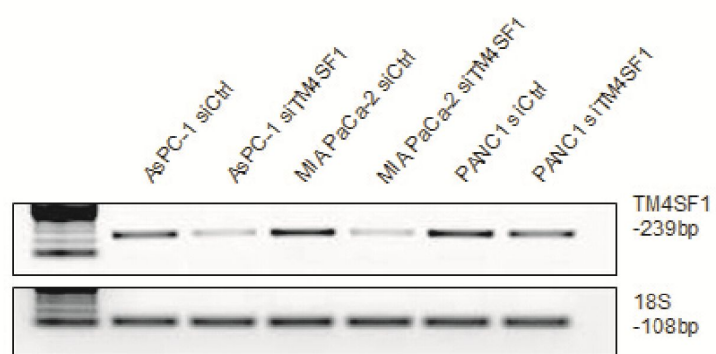

**Fig S1**

Supplement: S1 Fig — The cell lines were transiently transfected with siControl or siTM4SF1, and mRNA was isolated from them after 48 hours. (PDF) [file pone.0144969.s001.pdf]
